# Institutional Notes and News

**Published:** 1910-07-02

**Authors:** 


### Institutional Notes and News.

**Cottage Hospital, Ulverston.**

The chief fact of importance which was made public at the annual meeting of the Ulverston and District Cottage Hospital, Lancashire, was the astonishing financial success of the hospital parade. One-third of the hospital's annual revenue—in other words, no less than £300—was gained by it. We are not surprised that the report of the chairman of committees, Mr. Edmund Mackereth, gives full-measured praise to the Secretary of the Parade organisation and to his assistants. As his report says: "Does the public realise the hours of strenuous labour and worry accomplished by the said secretary and his assistants in bringing this pageant into working order and making it the success it is? There are men with tablets erected to their memories who have done less good voluntary work than those connected with the Parade in furthering the interests of our institution." It is perfectly true, and not only of Ulverston. In spite of these efforts, however, the year ended with a debt of £222, a satisfactory reduction of the £358 overdraft with which it started. The number of patients treated was 204, the total revenue £954, and the total expenses £818. A new bathroom is being made, the old one "with the bath sunk six inches in a concrete floor," having been converted into a room for washing all the articles used by the patients. This is a genuine improvement, but the members of the Parade committee are the heroes of the year.

**Cobham Cottage Hospital.**

There was no hesitation in adopting the sixth annual report of the Cobham and District Cottage Hospital. Work and income alike were felt satisfactory. The work had consisted of the treatment of 146 patients, an increase of thirty-nine over those received in the previous year, and this increase was reflected also in the income, which had risen to £595; that is to say, £30 more than the expenditure. The hospital possesses a reserve fund which amounts at present to £205, and is anxious particularly to increase it, so that the present site may be purchased, and at any rate a beginning made with the scheme for a new women's ward, which is much wished for. It is felt that the less delay there is in this matter the better, for the population of Surrey as a county, and perhaps especially that of Cobham, is increasing steadily, and therefore the demand on the hospital is likely to be more and more strenuous as time goes on. We understand that a special effort is being made this year to increase the reserve, which will be directed first to those who have not subscribed yet in any form to the hospital. The annual meeting was presided over by Mr. J. J. Morrish, on whose motion the report was adopted.

**New Hospital for Women, Euston Road.**

Last week an occasion of sincere interest to the New Hospital for Women, Euston Road, and particularly to women doctors, took place. This was the presentation to the institution of a portrait of the late Miss Charlotte Ellaby, M.D., the work of Mr. Basil Martineau. It will be remembered that Miss Ellaby took her degree of M.D. at Paris in 1884, and went out immediately afterwards to Bombay. After organising the eye department of the Cama Hospital for Women and Children there, and carrying on with real success the out-patient department, she resigned and returned home to devote her whole time to her favourite study (opthalmology). Miss Ellaby then became an L.S.A., for the Society had just opened its doors to women workers, and it was in 1890, we believe, that her close connection with the New Hospital for Women began. She turned her attention to the foundation of an eye department, and became the first ophthalmic surgeon at the New Hospital. Miss Ellaby remained till bad health, consequent, as she believed, on a trip to India in 1894 for professional purposes, caused her to resign her post, when she was appointed consulting ophthalmic surgeon to the hospital. Sir Frederick Lely presented the portrait, and expatiated on the energies by which Miss Ellaby had triumphed over the odds which beset the women doctors of her generation, and added that her work and that of her friend, Mrs. Pechey-Thompson (Dr. Edith Pechey), had borne useful fruit in the East. Mr. Pollock, the chairman of the New Hospital Committee, accepted the portrait, and said how valuable Miss Ellaby's work had been during the sixteen years she had held the post of first ophthalmic surgeon. The Memorial Fund is still open, any further sums received being devoted to helping poor ophthalmic patients.

**Middlesex Hospital.**

No apter example of the practical way in which the Royal Family regards the duty of personal service could be found than the energetic discrimination with which its various members have helped on the appeal of the Middlesex Hospital. Prince Francis of Teck, the Chairman, has his reward in the letter from her Majesty the Queen, which appears on another page. Hospital workers who have followed the campaign of this appeal—a campaign genuine from the fact of the many competing interests now distracting the public—will have gained more than a hint of what personal service means, and of the manner in which personal service can be made most useful.

**Royal Free Hospital.**

It is not often that hospitals appear as prosecutors, and therefore the following odd case is not without interest. Early in June this year a certain Mr. John Leveson, aged twenty, and a cabinetmaker by trade, applied for admission at the Royal Free Hospital. He was admitted, examined the next day, and, though the doctor thought there was nothing wrong with him, remained in the wards for three days, complaining that he suffered from dizziness and other products of a recent accident. The letter Mr. Leveson brought with him was first believed to have been written by a medical man, but no such person as the signatory was found in the Medical Directory. The result was a prosecution on the part of the governors of the hospital, alleging that the quondam patient had obtained medical care by false pretences. Detective Butt stated in his evidence that the accused "had been in and out of hospitals for the past three years under false pretences." The magistrate said that there was not enough evidence to convict, and the prisoner was discharged. It is noteworthy that of all the hospitals thus abused only the Royal Free, it appears, has taken the matter into court. It would be interesting to know how often these cases occur, and whether the policy of keeping quiet about the matter is adequate? A comparison of figures on this point in the various hospitals would be worth making. Such odd instances are often surface indications of evils fairly widespread. The above case adds yet another to many forms of "hospital abuse," though Mr. Leveson was not convicted.

